# Reprocessing 16S rRNA Gene Amplicon Sequencing Studies: (Meta)Data Issues, Robustness, and Reproducibility

**DOI:** 10.3389/fcimb.2021.720637

**Published:** 2021-10-21

**Authors:** Xiongbin Kang, Dong Mei Deng, Wim Crielaard, Bernd W. Brandt

**Affiliations:** ^1^ Department of Preventive Dentistry, Academic Centre for Dentistry Amsterdam (ACTA), University of Amsterdam and Vrije Universiteit Amsterdam, Amsterdam, Netherlands; ^2^ Genome Data Science, Center for Biotechnology, Faculty of Technology, Bielefeld University, Bielefeld, Germany

**Keywords:** 16S rRNA gene sequencing, reprocessing, rarefying, robustness, reproducibility, microbiome

## Abstract

High-throughput sequencing technology provides an efficient method for evaluating microbial ecology. Different bioinformatics pipelines can be used to convert 16S ribosomal RNA gene amplicon sequencing data into an operational taxonomic unit (OTU) table that is used to analyze microbial communities. It is important to assess the robustness of these pipelines, each with specific algorithms and/or parameters, and their influence on the outcome of statistical tests. Articles with publicly available datasets on the oral microbiome were searched for, and five datasets were retrieved. These were from studies on changes in microbiota related to smoking, oral cancer, caries, diabetes, or periodontitis. Next, the data was processed with four pipelines based on VSEARCH, USEARCH, mothur, and UNOISE3. OTU tables were rarefied, and differences in α-diversity and β-diversity were tested for different groups in a dataset. Finally, these results were checked for consistency among these example pipelines. Of articles that deposited data, only 57% made all sequencing and metadata available. When processing the datasets, issues were encountered, caused by read characteristics and differences between tools and their defaults in combination with a lack of detail in the methodology of the articles. In general, the four mainstream pipelines provided similar results, but importantly, P-values sometimes differed between pipelines beyond the significance threshold. Our results indicated that for published articles, the description of bioinformatics methods and data deposition should be improved, and regarding reproducibility, that analysis of multiple subsamples is required when using rarefying as library-size normalization method.

## Introduction

The development of massively parallel sequencing technologies made rapid sequencing of hundreds of samples at unprecedented depth possible (Schuster, 2008; Caporaso et al., 2011). This enabled researchers to apply 16S rRNA gene amplicon sequencing to analyze the composition and dynamics of complex microbial communities in depth (Woo et al., 2008). In the past decade, this has provided insights into diverse microbial communities, ranging from the ocean microbiome (Moran, 2015; Sunagawa et al., 2015; Mestre et al., 2018) or the soil microbiome (Fierer, 2017; Bahram et al., 2018; Delgado-Baquerizo et al., 2021; Xun et al., 2021) to the human microbiome (Turnbaugh et al., 2007; NIH HMP Working Group et al., 2009; Crielaard et al., 2011; Cho and Blaser, 2012; Gilbert et al., 2018).

To date, multiple approaches have been developed to process 16S rRNA gene amplicon sequencing data (Lemos et al., 2017). The most widely used software tools are USEARCH (Edgar, 2010), VSEARCH (Rognes et al., 2016), QIIME (Caporaso et al., 2010) [succeeded by QIIME 2 (Bolyen et al., 2019)], and mothur (Schloss et al., 2009). In addition, interest has grown in high-resolution clustering and error-correction of the sequences provided by tools, such as DADA2 (Callahan et al., 2016) and UNOISE (Edgar, 2016b). During the last years, many other pipelines combining different tools have been developed, such as OCToPUS (Mysara et al., 2017), FROGS (Escudié et al., 2018), PEMA (Zafeiropoulos et al., 2020), AmpliconTagger (Tremblay and Yergeau, 2019), Natrix (Welzel et al., 2020), and the MicrobiomeAnalyst platform (Chong et al., 2020). Conceptually, the processing pipelines are similar and can be divided into several steps: (1) paired-read merging; (2) quality filtering; (3) chimera removal; (4) clustering into operational taxonomic units (OTUs); and (5) taxonomic classification. After construction of the OTU table, researchers proceed to analyze the microbial composition and diversity of the microbial communities and to further interpret biological phenomena, for example, the relationship between obesity and gut microbiota (Komaroff, 2017).

However, algorithms and/or parameters in different processing pipelines often differ. So far, there is no single gold-standard pipeline to produce an OTU table (or higher-resolution count table), which means that both different tools and different parameters for the same step are being used in different pipelines.

Many existing processing steps have been evaluated, such as the influence of chimera checking methods (Edgar, 2016a; Mysara et al., 2017), denoising methods (Bonder et al., 2012; May et al., 2014), and clustering methods on the OTU table (Bonder et al., 2012; May et al., 2014; Westcott and Schloss, 2015; Mysara et al., 2017; Westcott and Schloss, 2017). Another study has assessed robustness and reproducibility of clustering methods on OTUs, while varying clustering thresholds (Schmidt et al., 2015). In addition, entire clustering or denoising pipelines have also been compared (Westcott and Schloss, 2015; Mysara et al., 2017; Nearing et al., 2018; Tremblay and Yergeau, 2019; Prodan et al., 2020). Several of these studies have shown in detail that both the number and composition of OTUs, from the same dataset, depend on the selected methods.

Here, we focused on the robustness of “final” results, which means a conclusion drawn from the same sequencing data is concordant among different processing pipelines [*cf*. (Schloss, 2018)]. We aimed to evaluate this using several published 16S rRNA gene amplicon studies and different mainstream pipelines. We are specifically not evaluating differences in the OTU tables themselves. We, and several others, have done that in the past and refer the reader interested in that to the articles cited above. While different pipelines likely result in different OTU tables due to their distinct algorithms and parameters, (biological) conclusions should rather not change. For example, if microbial profiles differ (significantly) between cases and controls, this should rather not depend on the pipeline. Thus, the aim is to look into statistical conclusions based on the analyses of the microbial profiles originating from several pipelines run on the same dataset.

To this end, four different pipelines based on VSEARCH, USEARCH, mothur, and UNOISE3, which are extensively used for 16S rRNA gene sequence data processing, were implemented; and publicly available datasets were retrieved and processed with these pipelines. Our aim is not to perform an exhaustive comparison of available pipelines. VSEARCH (Rognes et al., 2016) can be seen as an open-source reimplementation of USEARCH (Edgar, 2010). Since VSEARCH is used as a replacement for USEARCH, both tools were included as to see how their differences affect the final outcome. In addition, mothur (Schloss et al., 2009) was chosen as an often-used pipeline with an excellent SOP. Finally, UNOISE3 was selected as an example of a denoising method. It was found that UNOISE3 “showed the best balance between resolution and specificity” (Prodan et al., 2020).

Using the resulting OTU tables, differences in microbial α-diversity and β-diversity between groups within a study were evaluated and the results (P-values) compared among the pipelines, using *exactly* the same dataset. Since random subsampling is often used, we also evaluated reproducibility of results: a collection of subsampled OTU tables was generated as to compare the distribution of P-values within and between the pipelines. P-values are used here to illustrate differences among pipelines and should not be (mis)used to conclude about scientific importance (Baker, 2016; Wasserstein et al., 2019).

## Materials and Methods

### Dataset Search

Articles on the oral microbiome were searched for, and their respective datasets were retrieved. To limit the influence of the 16S rRNA region, this study only searched for datasets using the V4 16S rRNA region, published during the past 5 years (Illumina MiSeq sequencing). Both sequencing and metadata had to be publicly available. Initially, articles with deposited datasets were searched for using the NCBI website as this hosts both PubMed and the Sequence Read Archive (SRA). PubMed search results were linked to SRA using LinkOut (not possible anymore in the new PubMed). However, many articles that deposited data in the SRA, with article title and DOI, were lost in this process due to incomplete linking between these databases. Therefore, studies were searched for using Google Scholar with the following query (February 9, 2019): intitle:oral 16S “V4 region” OR “V4 variable region” OR “V4 hypervariable region” “accession OR SRA.” The final papers were screened on reported P-values for comparisons: at least one test on the microbiome data had to report a P-value between 0.0001 and 0.05. Finally, sequencing data and metadata were downloaded from the NCBI.

### Pipelines

**Figure 1:**
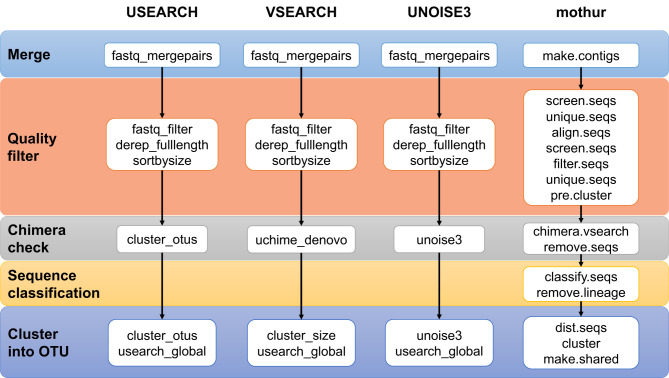
Overview of the four pipelines compared in this study. See **Supplementary Table 1** for details.

Four different processing pipelines were built to produce OTUs tables: a mothur pipeline [version 1.41.3], a VSEARCH [version 2.11.0-linux-x86_64], a USEARCH [version 11.0.667_i86linux32], and a UNOISE3 [version 11.0.667_i86linux32] pipeline. **Figure 1** presents an overview of the four pipelines, and **Supplementary Table 1** lists their details. In general, each pipeline used the standard commands with either default or otherwise well-accepted parameters. For mothur, we followed the MiSeq Standard Operation Procedure (https://www.mothur.org/wiki/MiSeq_SOP, d.d. 2019-01-24). We only changed the value of maxlength in screen.seqs from 275 to 258 as the V4 region has a small length variation and as to use the same value in all four pipelines. In the VSEARCH, USEARCH, and UNOISE3 pipelines, the reads were merged and quality-filtered per sample and then combined into one file. In the (32-bit) USEARCH/UNOISE3 pipelines, (64-bit) VSEARCH was used to dereplicate these quality-filtered sequences. Since the read lengths in the different studies differed (250 nt, but 150 nt in dataset 4 only), during merging a maximum of 10% mismatches in the overlap region was used.

### Analysis of OTU Tables

Statistical analyses were conducted with R [version 3.5.1, (R Core Team, 2018)] and the R packages microbiome [version 1.4.2, (Lahti and Shetty, 2017)], phyloseq [version 1.26.0, (McMurdie and Holmes, 2013)], and vegan [version 2.5-4, (Oksanen et al., 2019)]. The Mann-Whitney test was applied to test for differences in α-diversity (Shannon diversity index) between two different sample types, while differences in β-diversity were assessed using PERMANOVA (adonis, Bray-Curtis distance, 9999 permutations). Spearman’s rank correlation coefficient was used to correlate the Shannon diversity index between different pipelines. To evaluate the similarity between OTU tables (mothur only), a Procrustes Analysis and Mantel test were conducted with QIIME v1.9.1 (Caporaso et al., 2010) using the Bray-Curtis distance and 999 permutations.

Random subsampling was used to normalize unequal sample depth (library size). The subsampling depth for each dataset was determined such that most samples remained in the analysis, while adhering to minimum of around 2,000 reads/sample. In addition, as sample depths depend slightly on the pipeline, the subsampling depth was chosen such that the OTU tables from the different pipelines contained the same samples. To assess the reproducibility of statistical tests, 1,000 random subsamples of the same OTU table were analyzed.

## Results

Different publicly available datasets on the oral niche were searched for and processed with the VSEARCH, USEARCH, mothur, and UNOISE3 pipelines. During processing, different issues were encountered with specific datasets and pipelines that had to be addressed first. Next, the influence of the pipelines on diversity comparisons and reproducibility of results were evaluated.

### Dataset Search

The literature search returned 60 articles of which, upon inspection, many did not satisfy our criteria (see *Dataset Search* in *Materials and Methods*). Out of 53 articles that included an accession number to, for example, NCBI’s SRA or the European Nucleotide Archive, 45 studies actually deposited the raw data, while only 30 included the metadata in the database or in the article. Finally, 11 studies remained that used the V4 16S rRNA region and were related to the oral niche (19 studies were excluded: 14 studies used the V3-V4 region, 1 study used the V1-V2 region, 1 study the V1-V3 region, 3 studies on gut only). Based on screening with the P-value criterion, five oral microbiome studies were selected from these 11 studies. This criterion was used to restrict our analyses, since results would unlikely differ for more extreme P-values.

These datasets passing all criteria were the following. Dataset 1 (Stewart et al., 2018) was a study on the effects of tobacco smoke and electronic cigarette vapor exposure on the oral and gut microbiota. Dataset 2 (Schmidt et al., 2014) was on the relation between oral cancer and oral microbiota, and dataset 3 (Gomez et al., 2017) on the influence of host genetics on caries using monozygotic and dizygotic twins. Dataset 4 (Xiao et al., 2017) studied the impact of diabetes on the oral microbiota using mice, while dataset 5 (Chen et al., 2018) investigated the effects of periodontitis and its treatment on oral microbiota. **Table 1** shows an overview of these datasets. The raw read lengths were 250 nt, and, for dataset 4 only, 150 nt.

**Table 1:** Overview of the five used datasets.

Dataset	Accession	Study size	Selected depth	Species	Sample type	Sample types
Dataset 1 (Stewart et al., 2018)	PRJNA413706	90	9,500	Human	Saliva, buccal swabs, feces	Electronic cigarette users, tobacco smokers, and matched controls
Dataset 2 (Schmidt et al., 2014)	PRJEB4953	83	22,000	Human	Buccal swabs	Oral cancer, precancer, and healthy controls
Dataset 3 (Gomez et al., 2017)	PRJNA383868^*^	484	2,800	Human	Plaque	Twins, healthy or with enamel or dentin caries
Dataset 4 (Xiao et al., 2017)	SRP108800	81	1,900	Mouse	Saliva, feces	Normoglycemic, diabetic, and diabetic IL-17A antibody-treated mice
Dataset 5 (Chen et al., 2018)	SRP075100	238	7,900	Human	Saliva, plaque	Chronic periodontitis patients and periodontally healthy adults

Dataset 1 consisted of 90 samples from 30 participants: 10 tobacco smokers (TS), 10 electronic cigarette (EC) users, and 10 non-smoking controls. Fecal, saliva, and buccal swab samples were collected from each individual. Dataset 2 contained 83 samples divided over three groups: oral cancer (Cancer, n=21; Contralateral normal, n=19), precancer (Precancer, n=13; Contralateral normal, n=11), and healthy (lateral tongue, n=9; Floor of mouth, n=10) persons. For dataset 3, metadata included 485 dental plaque samples (484 twins and 1 singleton; dizygotic n=280; monozygotic n=205), while this singleton (1061.1_RD1) was not present in the SRA (^*^accessions: SRR5467515–SRR5467785 and SRR5467788–SRR5468062). In addition, eight samples did not include zygotic information. Finally, 271 dizygotic (DZ) and 205 monozygotic (MZ) samples remain. Samples from MZ/DZ twins were compared according to caries status: without dental caries (Health) or enamel/dentin caries, or treated caries. Dataset 4 contained 81 samples (45 oral swab samples and 36 fecal samples, which should be oral swab samples). Normoglycemic (Pre−Diab NG) and diabetes-prone mice (Pre−Diab DB) before and after (Diab NG; Diab DB) the development of hyperglycemia were sampled. In the oral swab samples, Normal refers to mice that received oral bacterial from normoglycemic mice, Diabetic to mice that received oral bacterial from diabetic mice, and Diabetic + IL17 to the mice treated with IL-17A antibodies and oral bacterial from diabetic mice. Dataset 5 comprised 238 samples collected from periodontal healthy individuals and chronic periodontitis patients: D1P (diseased/pre-treatment plaque n=96), D2P (diseased/post-treatment plaque n=19), HP (healthy plaque n=42), D1S (diseased/pre-treatment saliva n=45), D2S (diseased/post-treatment saliva n=18), and HS (healthy saliva n=18).

### Data Processing

Although it seemed straightforward to process the retrieved sequence data with one of the pipelines, several unexpected issues were encountered that were related to the sequence data in combination with a specific pipeline. The read pairs of dataset 2 could not be merged by VSEARCH and dataset 4 lost 57% sequences in the mothur pipeline. In addition, mothur could not cluster the sequences of datasets 3 and 4 into OTUs on our compute nodes (64 Gb RAM, 16 core CPU: E5-2650 v2 2.60 GHz) within the imposed time limit of 120 h. Finally, the deposited data of dataset 5 consisted of already merged read pairs.

Therefore, the processing of these datasets was slightly altered to address these issues. In dataset 2, the tail of the reverse reads contained approximately 100 bp low-quality bases (Q <= 2) preventing the read pairs to be merged by VSEARCH. However, USEARCH merged these reads, because the used version automatically trims these low-quality tails (Q <= 2) before merging (Q <= 2, min. length 64 nt). Therefore, we pre-filtered dataset 2 using Trimmomatic v.0.38 (Bolger et al., 2014) with “TAIL:3 MINLEN:64” and used this filtered data as input for all pipelines.

From dataset 4, many sequences were removed after the mothur screen.seqs command on the aligned sequences, in which the sequences are required to span at least the V4 region (from 1968 to 11550) in the alignment. Manual inspection showed that many sequences ended one position early and that the first base call after the V4 (806R) reverse primer was absent. Therefore, for dataset 4 only, the value of the end parameter in this screen.seqs command was changed from 11,550 to 11,549 to avoid losing 57% of the sequence data.

In addition, both datasets 3 and 4 contained many singletons. This caused the OTU clustering to fail in mothur. Therefore, singletons were removed from datasets 3 and 4 in the mothur pipeline (split.abund, cutoff=1). For dataset 3, it was also possible to generate an OTU table with cluster.split (taxlevel=2, cutoff=0.03). To evaluate the difference between these two OTU tables (i.e., from cluster.split or singletons removed), they were compared. Spearman’s correlation of the Shannon diversities (R = 0.9895, P-value < 2.2e-16), Procrustes Analysis (M^2 = 0.01; p < 0.001), and the Mantel test (r = 0.98837, P-value = 0.001) showed that the OTU tables were very similar. Since dataset 4 could not be processed with cluster.split within the wall-time limit of 120 h, no comparison could be made and the dataset with singletons removed was used.

After the modifications described above, all five datasets were processed with all four pipelines. The total numbers of raw, merged, quality-filtered reads, and reads mapped to the OTU table are summarized in **Supplementary Table 2**. For dataset 4, the OTU table from mothur contained only 69% of sequences of the table from the other pipelines. This turned out to be caused by the removal of non-bacterial sequences (chloroplast, mitochondria, unknown, archaea, eukaryota) in the SOP mothur pipeline. Since only dataset 4 contained many non-bacterial sequences, for all pipelines applied to dataset 4, OTUs classified as non-bacterial were removed as to make a fair comparison.

### Robustness of Results

For each of the datasets, OTU tables were generated by the different pipelines. The fraction of quality-filtered mapped reads represented in the OTU table was similar (datasets 1, 2, 3, 5 combined: average 0.95, standard deviation 0.023; dataset 4: average 0.60, standard deviation 0.0018; **Supplementary Table 2**). However, within a dataset, the number of OTUs depended on the pipeline, where the mothur pipeline generated most OTUs. Next, OTU tables were rarefied to avoid the influence of sample depth differences within one dataset (**Table 1**), and the general similarity among these tables from the pipelines was compared using Spearman’s rank correlation of the Shannon diversity index. All correlations were high, ranging from 0.94 to 1.0 (P-values < 2.2e-16).

To evaluate the robustness of α-diversity results, the Shannon diversity indices of two different sample types present in the dataset (the original study) were compared for the different pipelines (Mann-Whitney test; single subsampled OTU table). A heatmap (**Supplementary Figure 1A**) shows the resulting P-values, most of which were similar to the original results. Since conclusions, thus biological inferences, are more likely to depend on data processing details when P-values are closer to the significance threshold, we zoomed in on the eight comparisons that had a P-value below 0.05.

**Figure 2:**
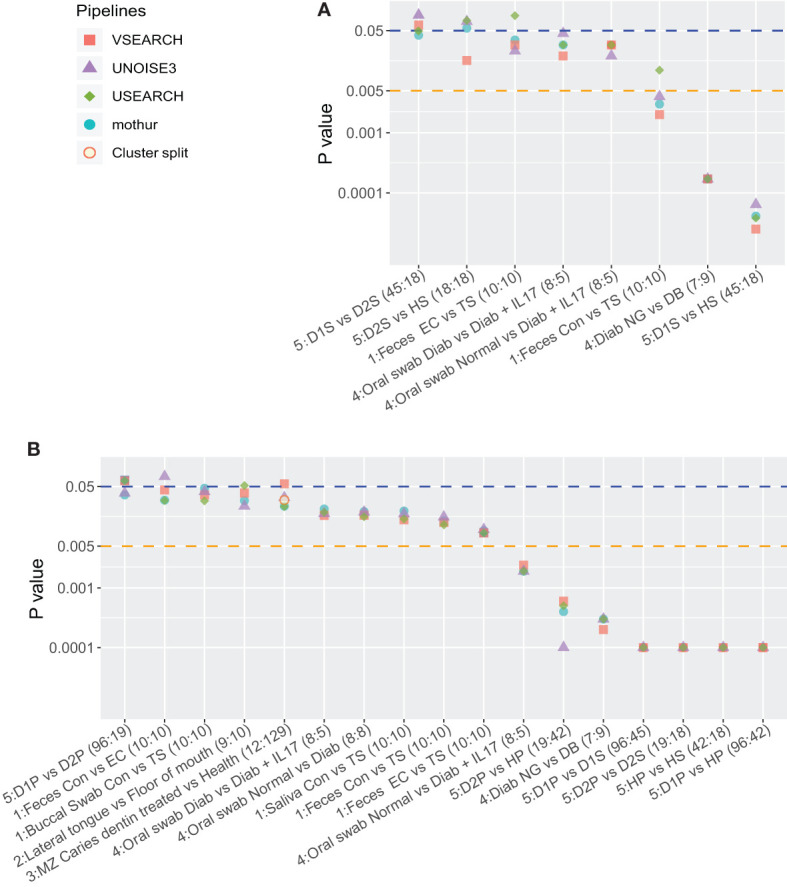
Overview of P-values of the five datasets sorted on decreasing average P-value. The first number before the colon indicates the dataset. Cluster.split is an alternative mothur pipeline used only for dataset 3. **(A)** P-values of Mann-Whitney tests on the Shannon diversity index (α-diversity) and **(B)** P-values of PERMANOVA (Bray-Curtis distance, β-diversity) tests between two sample types. In **(A)**, at a threshold of 0.05, once VSEARCH differed from the other pipelines, once USEARCH, and once mothur and USEARCH differed from VSEARCH and UNOISE3. At a threshold of 0.005, there was one conflict (USEARCH). In **(B)** at 0.05, there were four conflicts: once mothur and UNOISE3 were the same, but differed from USEARCH and VSEARCH, once UNOISE3, once USEARCH, once VSEARCH.

Using the significance threshold of 0.05, five comparisons resulted in identical biological conclusions, while there were three conflicts between the four pipelines (**Figure 2A**). Recently, studies proposed to lower the significance threshold to 0.005, which would “immediately improve the reproducibility of scientific research” (Benjamin et al., 2018; Ioannidis, 2018). When the significance threshold was lowered to 0.005, one conflict remained.

**Figure 3:**
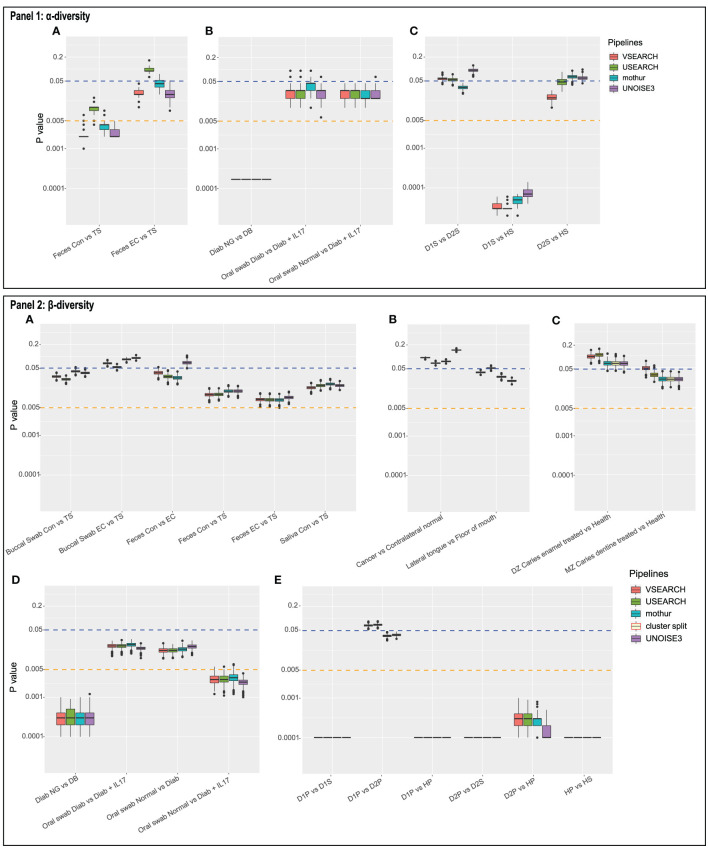
Panel 1 shows the distribution of P-values of Mann-Whitney tests on the Shannon diversity index between the indicated two sample types for 1,000 random subsamples in **(A)** dataset 1, **(B)** dataset 4, and **(C)** dataset 5. Panel 2 shows the distribution of P-values of PERMANOVA (Bray-Curtis distance) tests for 1,000 random subsamples in datasets 1 to 5 **(A–E)**. Cluster.split is an alternative mothur pipeline used only for dataset 3 **(C)**.

Similarly, as to assess the robustness of between-group differences, the microbial profiles of the two groups of sample types were subjected to PERMANOVA (Bray-Curtis distance; **Supplementary Figure 1B**). In most cases, the P-values were similar among the different pipelines and to the original results. Of the 28 comparisons (**Supplementary Figure 1B**), 17 groups had P-values below 0.05 (**Figure 2B**). Similar to the α-diversity tests, lowering the significance threshold improved robustness. However, at any significance threshold, differences between pipelines, here on the *same* data, can appear (**Figure 3**).

In some cases, published results differed from ours, which can also be related to a different distance metric used (datasets 1 and 2 did not use Bray-Curtis). As an example, we take dataset 1, which was processed by the authors using USEARCH (Stewart et al., 2018). In our study, the P-values for the fecal microbiota of controls (Con) *versus* electronic cigarettes (EC) users slightly depended on the pipeline (P-value range: 0.03–0.07). However, the much higher P-value reported by the authors was related to the weighted UniFrac distance metric. Indeed, when using the OTU table provided by authors, all PERMANOVA (Bray-Curtis) results became very similar (**Supplementary Figure 1B**).

### Reproducibility of Results

This study also evaluated the reproducibility, defined here as “re-analysis with exactly the same pipeline and same dataset supports an identical conclusion.” To this end, each OTU table was subsampled 1,000 times, and statistical tests were done as above, for each of the 1,000 tables, thus providing 1,000 P-values (boxplots in **Figure 3**). Since the P-value ranges for a given pipeline can cross a significance threshold (e.g., **Figure 3-1B**) or can be large (**Figure 3-2D**), care should be taken with reporting results (publication bias).

Subsampling datasets with a large standard deviation in sample depths can lead to a larger variation in test results. For example, within dataset 4, the P-value distribution for UNOISE of the first comparison (**Figure 3** **-2D**; Diab NG *vs* DB; range: 0.0001–0.0012) differed from other three (e.g., Oral swab normal *vs* Diabetic+IL17: 0.001–0.004). In the first comparison, the median depths of the groups differed a lot (7,666 *vs* 52,512); in the latter, they were much closer (11,387 *vs* 11,572). Thus, when subsampling, results can show more variation since random subsamples vary more when subsample depth is low compared to the sample depth and/or when there is a bias in sample depth between groups. However, this argument does not hold for, for example, dataset 5: D2P *vs* HP and D1P *vs* HP. Here, all three sample groups have very similar medians. However, variability can also be caused by biological differences as well as sample size differences (D2P: 19, D1P: 96, HP: 42 samples). As to exclude biological and other differences between samples, dataset 1 was subsampled at a lower depth to illustrate the increased variability using the same data (**Supplementary Figure 2**). Not surprisingly, a lower subsampling depth results in higher variability of test results.

## Discussion

It is difficult to make research sufficiently transparent and reproducible, especially in interdisciplinary fields such as microbiome studies (Schloss, 2018). In this study, we evaluated the robustness and reproducibility of 16S rRNA gene amplicon studies using four mainstream pipelines.

It was not straightforward to reprocess or reproduce results of these studies. During our literature search, we encountered many articles with no or incomplete data availability, even though an accession number was provided: only 57% provided sequencing data and metadata. In addition, while correct and complete descriptions of methods and metadata are crucial, they are often not provided in sufficient detail. Although unclear descriptions of processing methods were not such an issue in this work, since we used our own pipelines, phrases like “reads were quality-filtered” or “clustered using UCLUST” are much too imprecise.

Due to (implicit) differences between tools used for the pipelines, we sometimes had to adapt a pipeline to the data at hand (see *Data Processing* in *Results*). For example, in dataset 4, about 35% of the sequences was taxonomically classified as chloroplast (40% as non-bacterial). However, in the corresponding article (QIIME 1 was used), we did not explicitly find that these sequences were removed, although that seemed to be the case (**Supplementary Figure 3**). Clearly, each dataset requires specific steps, also with respect to quality filtering, and it is important to be aware of differences among tools (even related ones as USEARCH and VSEARCH, or different versions of the same tool).

The pipelines resulted in a different number of OTUs, which is not surprising. Nearing et al. (2018) reported that several denoising pipelines largely influenced α-diversity (observed OTUs) and possibly impact results based on α-diversity, while the weighted β-diversity metrics (Bray-Curtis, weighted UniFrac) were very similar among different pipelines. When comparing the results of tests on diversity, i.e., the distribution of P-values between pipelines and within a pipeline (**Figures 2**, **3**), tests on α-diversity (Shannon) seem to show a larger variation than on β-diversity (Bray-Curtis, PERMANOVA).

Irrespective of the above, some differences related to tests on α-diversity were initially unexpected, such as between USEARCH and VSEARCH (e.g., Shannon diversity in **Figure 2A**, datasets 1 and 5). Since VSEARCH can be seen as an open-source USEARCH, it was hypothesized that this difference was caused mainly by the different method of chimera checking in these pipelines: USEARCH performs this during clustering, while with VSEARCH this was done before clustering (uchime_denovo). To analyze this, dataset 1 was processed with a VSEARCH pipeline in which the chimera-checking method was replaced by USEARCH (cluster_otus). Indeed, now the test results were more similar to those of USEARCH (**Supplementary Figure 4**). Thus, in this case, the results for Shannon diversity seem to be sensitive to chimera-detection methods. According to a previous study, different chimera-detection methods influenced the accuracy of clustering (May et al., 2014). The result of this study further demonstrated that differences in chimera-checking methods also affected robustness. For these datasets, DADA2, which has a different chimera-checking method, can also show differences due to false positive chimeras (Edgar, 2016b).

The ranges of P-values, based on the 1,000 subsampled OTU tables, sometimes exceeded a significance threshold. This showed that when OTU tables are rarefied, reproducibility can be affected. A P-value of 0.06 does not really differ from 0.04 [*cf*. (Halsey et al., 2015)], and larger differences occur using exactly the same data (**Figure 3**). At lower subsampling depth, with respect to the median sample depth of a group, and/or when depths have large standard deviation, reproducibility can decrease. Especially in such cases, given rarefying is the chosen normalization method, multiple randomly subsampled OTU tables should be evaluated, and the median P-value be used.

Here, rarefying, which is still very often used, was applied to normalize library size. The comparison of normalization methods was beyond the scope of this study, but we note that different methods are available [proportion, CSS, log-ratio, TMM, *cf*. Weiss et al. (2017)]. While McMurdie and Holmes (2014) stated rarefying should not be used to detect differentially abundant species and better be generally avoided, Weiss et al. (2017) later reported that rarefying itself seemed not to increase false discovery rates of many differential abundance-testing methods, and even lowered the false discovery rate when the average library size for groups differed a lot (~10×). While it is not straightforward which normalization method should best be used, even though data normalization methods now receive ample attention, we should not forget “subsampling” occurs several times during experimental procedures, ranging from biological sampling, dilution of DNA for amplicon PCR, to generating the equimolar mix for sequencing.

Irrespective of the normalization technique, care should be taken with PERMANOVA. As stated with its introduction (Anderson, 2001), calculating all possible permutations usually is unrealistic, considering computational time. However, increasing the number of permutations improves the precision of the P-value (Anderson, 2001). With a lower number of permutations (e.g., 999 instead of 9,999), the range of P-values (using same OTU table) increases, which can affect reproducibility. This then shows that the permutation space is too undersampled and the number of permutations should be increased (*cf*. page 37 in Anderson, 2001). Thus, PERMANOVA should be repeated as to check the P-value varies little.

This study did not evaluate the differences caused by the use of different diversity indices (e.g., species richness, Chao1 richness, Shannon index) or distance metrics [(weighted) UniFrac, Bray-Curtis, Jaccard], since these are different downstream choices. When evaluating results from published studies, we should remember that different α-diversity indices or β-diversity metrics can lead to different conclusions. However, here, the focus was on whether different conclusions would result from different amplicon processing pipelines.

Although QIIME 1 also was often used, it has not been supported since 2018, and we did not include it as to keep comparisons concise. In addition, based on previous studies, the default QIIME 1 pipeline has higher error rates due to chimeras and higher amount of spurious OTUs comparing with others (Mysara et al., 2017; Prodan et al., 2020). We, therefore, only compared VSEARCH, USEARCH, mothur, and UNOISE3 in this article as example pipelines, to limit variations and maintain focus, but note that QIIME 2 also supports a VSEARCH pipeline.

In general, results of the four pipelines were robust and reproducible, with some conflicts around the 0.05 threshold (**Figure 2**). The choice of 0.05 as P-value threshold was arbitrary, and it was proposed to lower the P-value threshold to 0.005 to “improve reproducibility of scientific research” among studies (Benjamin et al., 2018; Ioannidis, 2018). However, a different, sometimes related, pipeline for the *same* dataset (study) resulted in different P-values. Although we cannot conclude that a lower threshold should be used, we should keep in mind that P-values just below 0.05 may not be very robust or reproducible, and a lower threshold also comes at a cost (Di Leo and Sardanelli, 2020). Irrespective of the used thresholds, we recommend that real P-values are always reported (not as: “P<0.05”).

In our limited exploratory analysis, we did not find that clustering methods consistently differed to the denoising method. With the introduction of UNOISE, Robert Edgar stated, “I suggest you try both. If a biological conclusion is different, then you should worry that neither result is trustworthy” (Edgar, 2019). Yet, it is important to realize that using the same sequencing data, (1) results among pipelines can differ; (2) it will often not be straightforward to uncover why specific differences occur; (3) generally a single pipeline is used, so differences will remain unnoticed. In addition, other measures than P-values can be considered as these show large sample-to-sample variability and have other issues (Halsey et al., 2015; Wasserstein et al., 2019). Nevertheless, a discussion on the use P-values is beyond the scope of this article, and there is no consensus this subject (Halsey et al., 2015; Ioannidis, 2018; Ioannidis, 2019; Wasserstein et al., 2019; Di Leo and Sardanelli, 2020).

In summary, we conclude the following: Sequencing data and metadata should be properly deposited and journals should check if data have actually been made publicly available. Not surprisingly, different pipelines can lead to different statistical conclusions; thus, methods should be described in detail and include software versions, algorithms, and parameters used. While “the only direct protection [to the threat of selection bias] must come from standards for reproducible research (Ioannidis, 2019)”, microbiome research and its data processing highly depend on wet- and dry-lab technology, and even if standards would exist, they would repeatedly (need to) change (Amaral and Neves, 2021). This means that more care should be taken to share methods and (raw) data.

## Data Availability Statement

The datasets analyzed in this study can be found in the Sequence Read Archive (NCBI) or the European Nucleotide Archive (EBI) as indicated in **Table 1** or in their cited publications.

## Author Contributions

BB designed the research and assisted XK in the study design, data collection, and bioinformatics analysis. XK, DD, and BB wrote the draft. DD, WC, and BB reviewed the manuscript. All authors contributed in the preparation and finalization of the manuscript. All authors contributed to the article and approved the submitted version.

## Funding

XK is supported by the China Scholarship Council (CSC), grant number 201808440303.

## Conflict of Interest

The authors declare that the research was conducted in the absence of any commercial or financial relationships that could be construed as a potential conflict of interest.

## Publisher’s Note

All claims expressed in this article are solely those of the authors and do not necessarily represent those of their affiliated organizations, or those of the publisher, the editors and the reviewers. Any product that may be evaluated in this article, or claim that may be made by its manufacturer, is not guaranteed or endorsed by the publisher.
