# Supplementary material for: Reprocessing 16S rRNA Gene Amplicon Sequencing Studies: (Meta)Data Issues, Robustness, and Reproducibility
Source: Front Cell Infect Microbiol. 2021 Oct 21;11:720637. doi: 10.3389/fcimb.2021.720637 (PMC8566820; doi:10.3389/fcimb.2021.720637)
Supplement: Supplementary file 1 [file Table_1.pdf]

Supplementary Material

1 Supplementary Figures and Tables

1.1 Supplementary Figures

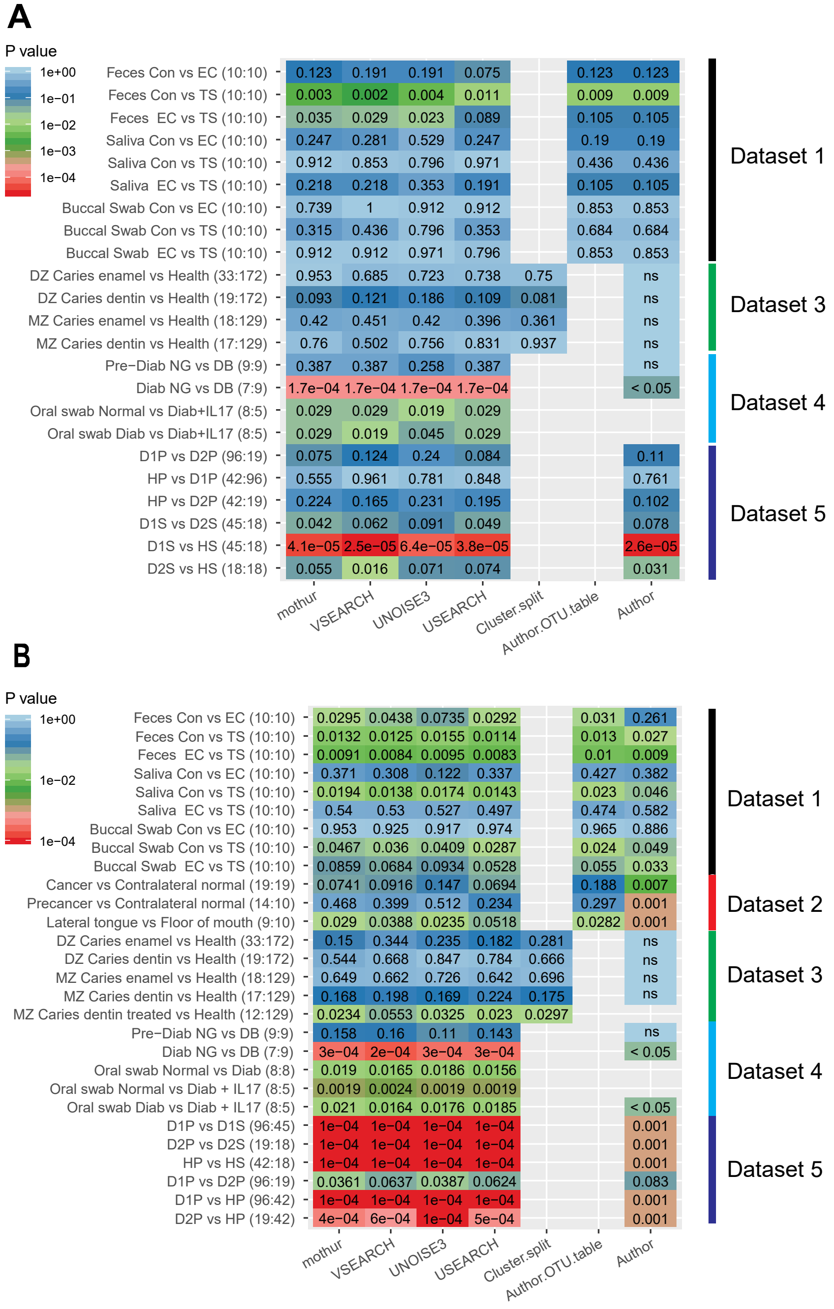

Supplementary Figure 1. (A) P-values of the Mann-Whitney test on the Shannon diversity index, and **(B)** P-values of PERMANOVA (Bray-Curtis) for the indicated two sample types.

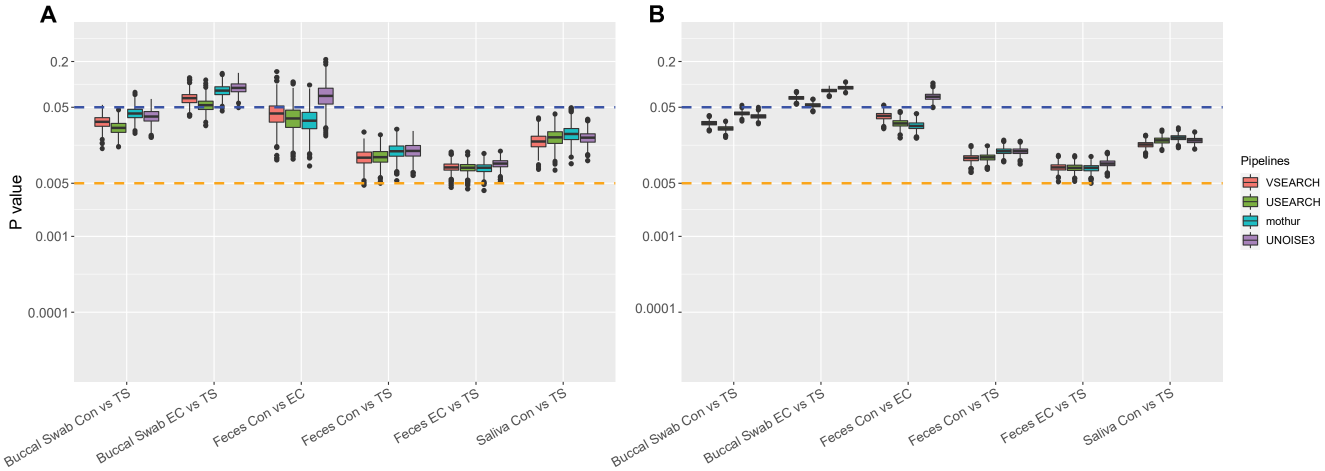

Supplementary Figure 2. The distribution of P-values from the PERMANOVA tests, both with 9999 permutations, on dataset 1 at a subsampling depth of **(A)** 1000 and **(B)** 9500. The latter is repeated here for overview and is identical to Figure 3-2A. Lower subsampling depth results in a larger variation of the test results.

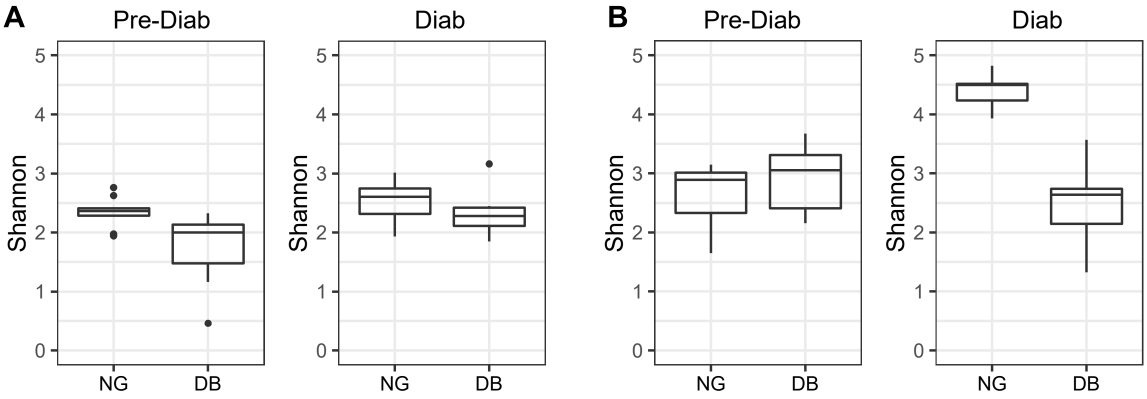

Supplementary Figure 3. Shannon diversity for type 2 diabetes-prone mice and lean normoglycemic controls in dataset 4 (VSEARCH). Non-bacterial sequences were **(A)** not removed or **(B)** removed. 9 normoglycemic mice (NG) and 10 (9 in B) diabetes-prone mice (DB) before hyperglycemia (left) and 8 (7 in B) normoglycemic and 9 diabetic mice after the diabetes-prone developed hyperglycemia (right). Subsample depth in A and B is 1900. Figure B is very similar to Figure 1A in (Xiao et al., 2017).

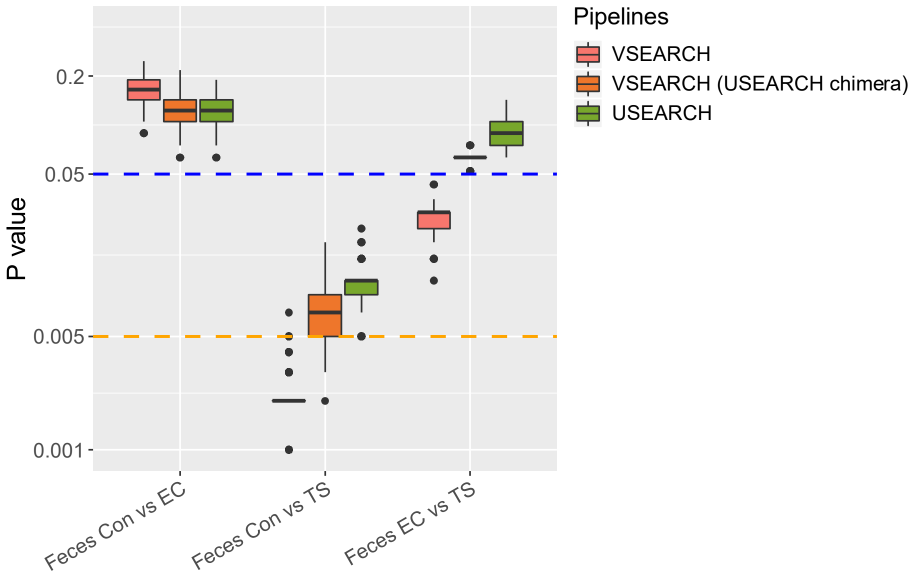

Supplementary Figure 4. The distribution of P-values of Mann-Whitney tests on the Shannon diversity index for the fecal samples of dataset 1 (1000 different random subsamples) for the VSEARCH and USEARCH pipelines. In "VSEARCH (USEARCH chimera)", chimeras indicated by USEARCH cluster_otus were removed. The difference in chimera detection method partly explained the different P-values for the Con *vs* TS and EC *vs* TS comparisons.

1.2 Supplementary Tables

Supplementary Table 1. Overview of evaluated pipelines, their commands and parameters.

<table>
<tr><th>Pipelines</th><th>Merging</th><th>Quality filtering</th><th>Dereplication</th><th>Chimera filtering</th><th>Clustering</th></tr>
<tr><td>VSEARCH</td><td>fastq_mergepairs, relabel,
fasta_width 0,
fastq_maxdiffs 100,
fastq_maxdiffpct 10,
fastq_minmergelen 249,
fastq_maxmergelen 258.</td><td>fastq_filter,
fasta_width 0,
fastq_maxee 0.5.</td><td rowspan="3">derep_fulllength
sortbysize: minsize 2.</td><td>uchime_denovo</td><td>cluster_size: id 0.97,
fasta_width 0,
usearch_global: id 0.97,
maxhits 1, maxrejects 64,
maxaccepts 8.</td></tr>
<tr><td>USEARCH</td><td rowspan="2">fastq_mergepairs, relabel,
fastq_pctid 90,
fastq_maxdiffs 100,
fastq_minmergelen 249,
fastq_maxmergelen 258.</td><td rowspan="2">fastq_filter,
fastq_maxee 0.5.</td><td>cluster_otus</td><td>cluster_otus
usearch_global: id 0.97,
maxhits 1, maxrejects 64,
maxaccepts 8.</td></tr>
<tr><td>UNOISE3</td><td>unoise3</td><td>unoise3
usearch_global, id 0.97,
maxhits 1, maxrejects 1024,
maxaccepts 128.</td></tr>
<tr><td>mothur</td><td>make.contigs</td><td colspan="3">screen.seqs, maxambig=0, maxlength=258, maxhomop=8; unique.seqs; align.seqs; screen.seqs: start=1968, end=11550; filter.seqs; unique.seqs; pre.cluster: diffs=2; split.abund: cutoff=1[#]; chimera.vsearch; remove.seqs; classify.seqs; remove.lineage;</td><td>dist.seqs cutoff=0.03
cluster cutoff=0.03
make.shared label=0.03
classify.otu label=0.03.</td></tr>
</table>

This parameter was only used for datasets 3 and 4. Full details on the commands and parameters can be found online: VSEARCH: https://github.com/torognes/vsearch, USEARCH/UNOISE: https://www.drive5.com/usearch/, mothur: https://mothur.org/wiki/miseq_sop/.

Supplementary Table 2: Read statistics for different steps in the pipelines for each dataset (XLSX).

References

Xiao, E., Mattos, M., Vieira, G.H.A., Chen, S., Correa, J.D., Wu, Y., et al. (2017). Diabetes enhances IL-17 expression and alters the oral microbiome to increase its pathogenicity. *Cell Host Microbe* 22**,** 120-128.e124. doi: 10.1016/j.chom.2017.06.014
